# Dr. James Marion Sims

**Published:** 1883-11

**Authors:** 


### DR. JAMES MARION SIMS.

This illustrious benefactor of his race is no more. He died Nov. 13, of heart disease, at his residence on Madison Avenue, New York. The civilized world will receive with sorrow the tidings of his death. No such man as Dr. Marion Sims has fallen in a century from the well-filled ranks of the medical profession; and it may be a century more, before, in God's providence, another like him shall stand up to take his place.

This unrivalled physician possessed all the attributes of true greatness, over and above the peculiar special gifts that enabled him to become the greatest surgeon of his times. Simple, natural, abounding in affection and good feeling for all around him, he was unpretentious as a child and loving as any woman.

Those who knew him best and enjoyed the intimacy of his confidence, were held bound to him, as it were, by hooks of steel. The magic of this social power was irresistible, and sprang directly from his perfect freedom from everything selfish or little. His friends adored him. He knew nothing of the worldly-wise, small policies of social or professional life, but to loath and despise them.

None who enjoyed the pleasure of his acquaintance could conceive the possibility that Marion Sims would ever, under any circumstances, decline the recognition of an old friend or neighbor, and whenever any of his neighbors whom he had known in his boyhood's home at Lancaster, S. C., came to New York city, as they sometimes did, it was his delight to take them to his sumptuous house on Madison Avenue and treat them like princes among his city acquaintances.

Not long before his death, a beloved relative of Mrs. Marion Sims, who was also of Lancaster, S. C., the Rev. J. Witherspoon, D.D., of New Orleans, visited him at his residence in New York. As soon as he recognized him (as it was recently related to the writer by Dr. W.), he ran out, in his usual whole-soul, open-arms way, to meet and greet him. And, after all were seated, leaning his head upon his wife, said: "Come, Cousin Jack, let us have a long talk about the old home and neighbors in Lancaster."

In this connection, it is said of him by a pupil* who knew him well in his hospital life in New York:

"So benignant, so entirely frank and cordial, his arms were always open and extended to the approaches of the young physician, and thousands of these, all over the world, recipients of his tender courtesies and substantial favors, will drop a tear to his precious memory."

In the pursuit of scientific and professional knowledge, he manifested the same child-like humility, modesty and courage that characterized him in his social relations. While still investigating a subject, and not yet sure of the truths involved, he was as teachable and impressible as any school-boy; but after a clear, unmistakable discovery of principles and facts, no power on earth could drive him from his conclusions or his course of practice thereon.

His aptitude for surgery appeared like an inspiration. Soon after his first appearance in Europe, the great surgeons both of England and the Continent, recognizing the mastery of his hand and genius, deferred to his superiority. The laity quickly discovered and rightly estimated his wonderful powers. Crowned heads and societies showered upon him in dazzling profusion, the splendid tokens of their confidence and admiration.

Never was there a professional character, who was more tempted and tried by the lavish blandishments of fortune. Yet his head was not turned. Like the truly great man that he was, he maintained a dignified simplicity and self possession to the last. At any stage of his life he would have preferred a plain country meal sur-

*Dr. E. H. Richardson, Cedartown, Ga.

rounded by his old friends and neighbors at Lancaster, to the most sumptuous of those to which he was so often invited in Europe.

We are told that at the dinners given by himself in Paris to distinguished guests and professional friends, while they were dining on the luxurious viands of the best French cookery, and quaffing delicious wines, the generous giver of the feast was, himself, making a hearty meal upon milk and potatoes.

Marion Sims was free from the minor bad habits tolerated in modern society. He used neither tobacco, wine nor strong drink, and the light of his joyous home was his happiness and stay.

When complimented on his professional success, he was accustomed to reply: "Yes, but I owe it all to my wife; she has been the inspirer, the guardian angel of my life."

Mrs. Marion Sims was, herself, the daughter of a distinguished physician and surgeon of Lancaster, Dr. Bartlett Jones, of precious memory to the good people of that sturdy community. His published operations were more than once reproduced in the *London Lancet*; and it is said that his illustrious son-in-law caught no small portion of his professional inspiration from witnessing Prof. Jones' operations while yet a boy.

Mrs. Marion Sims is a Presbyterian, and her husband, with herself, was brought up in the doctrines and under the sterling influences of that noble old church, which has done so much for the world, and for the intelligence and character of South Carolinians.

While Marion Sims made no public profession of religion, his views of practical piety, and of human responsibility, were in exact keeping with those traits of character which had made him great as a surgeon and a man.

On one occasion, in London, he and several American friends went to St. Paul's to hear Canon Farrar. After the sermon, while passing out of the cathedral, he remarked, "I could not understand him; he preached entirely over my head. I will go and hear the Salvation Army,—they preach more to my taste." He also added,

"If I were a preacher of the gospel, I should preach Jesus Christ and Him crucified."

He had no sympathy with the doctrine of evolution or any other form of skepticism or infidelity.

The great Christian doctrine of a special over ruling Providence was the subject of his peculiar belief and admiration. He was accustomed to say that if he possessed any skill as a surgeon or physician, it was the special gift of God, who gave him all his powers and guided him in all, of even the minutest and most trifling acts and incidents of his life.

It would seem superfluous to add, in conclusion, that Dr. Marion Sims was the model of a Southern gentleman. All who came in contact with him felt and acknowledged the benignity and gentleness of his genial nature. The old servants of his household in New York mourn for him as for a lost father or brother. The letter-carrier on Madison avenue said of him, "I have been delivering Dr. Marion Sims' letters for fifteen years, and during all that time I was received and treated by him as one worthy of his distinguished regard. There is no man like him."

He was the recipient of the Order of the Legion of Honor from the government of France. Belgium, Spain, Italy and Portugal conferred upon him similar distinctions.

But these honors, splendid as they are, are insignificant without the higher and more enduring glory which he has fairly won, of living in the hearts of millions who survive him, and of millions more to be born, who shall delight to keep fresh the memorials of him and all like him, who gave his life and best endowments for the benefit of his fellow-men.

Everything that this truly philanthropic physician ever discovered, invented or acquired in his divine art was freely given to the world. His writings are numerous, and their influence upon the medical world marks a new era in its history. It is highly probable that he lost more pecuniarily by their publication than he secured by their sale, but this was a matter of trivial importance to the author, so long as they went forth to alleviate human suffering and disease.

The country women, especially, of Dr. Marion Sims owe to his memory on the consecrated spot where repose his motal remains a monument, and the women of Europe a cenotaph, either in London or Paris, on both of which shall be inscribed a fitting testimonial of the achievements of this greatest of modern surgeons, and of the virtues of this rare, noble-hearted gentlemen and friend of mankind.

